# Current Innovations in Intraocular Pressure Monitoring Biosensors for Diagnosis and Treatment of Glaucoma—Novel Strategies and Future Perspectives

**DOI:** 10.3390/bios13060663

**Published:** 2023-06-18

**Authors:** Rubiya Raveendran, Lokesh Prabakaran, Rethinam Senthil, Beryl Vedha Yesudhason, Sankari Dharmalingam, Weslen Vedakumari Sathyaraj, Raji Atchudan

**Affiliations:** 1 Faculty of Allied Health Sciences, Chettinad Hospital and Research Institute, Chettinad Academy of Research and Education, Kelambakkam 603103, Tamil Nadu, India; rubijrf23@gmail.com (R.R.); lokesh@care.edu.in (L.P.); 2 Department of Pharmacology, Saveetha Dental College and Hospitals, SIMATS, Chennai 600077, Tamil Nadu, India; senthilrethinam.sdc@saveetha.com; 3 Regeneration and Stem Cell Biology Lab, Centre for Molecular and Nanomedical Sciences, International Research Centre, Sathyabama Institute of Science and Technology, Chennai 600119, Tamil Nadu, India; berylvedha.irc@sathyabama.ac.in; 4 Department of Biotechnology, College of Science and Humanities, SRM Institute of Science and Technology, Kattankulathur 603203, Tamil Nadu, India; sankari.d@ktr.srmuniv.ac.in; 5 School of Chemical Engineering, Yeungnam University, Gyeongsan 38541, Republic of Korea; 6 Department of Chemistry, Saveetha School of Engineering, Saveetha Institute of Medical and Technical Sciences, Chennai 602105, Tamil Nadu, India

**Keywords:** glaucoma, intraocular pressure, nanoparticles, lens, imaging, biosensors

## Abstract

Biosensors are devices that quantify biologically significant information required for diverse applications, such as disease diagnosis, food safety, drug discovery and detection of environmental pollutants. Recent advancements in microfluidics, nanotechnology and electronics have led to the development of novel implantable and wearable biosensors for the expedient monitoring of diseases such as diabetes, glaucoma and cancer. Glaucoma is an ocular disease which ranks as the second leading cause for loss of vision. It is characterized by the increase in intraocular pressure (IOP) in human eyes, which results in irreversible blindness. Currently, the reduction of IOP is the only treatment used to manage glaucoma. However, the success rate of medicines used to treat glaucoma is quite minimal due to their curbed bioavailability and reduced therapeutic efficacy. The drugs must pass through various barriers to reach the intraocular space, which in turn serves as a major challenge in glaucoma treatment. Rapid progress has been observed in nano-drug delivery systems for the early diagnosis and prompt therapy of ocular diseases. This review gives a deep insight into the current advancements in the field of nanotechnology for detecting and treating glaucoma, as well as for the continuous monitoring of IOP. Various nanotechnology-based achievements, such as nanoparticle/nanofiber-based contact lenses and biosensors that can efficiently monitor IOP for the efficient detection of glaucoma, are also discussed.

## 1. Introduction

Visual sensing is one of the most crucial requirements in a human’s life. The eye plays a vital role in connecting human beings with the external world [1,2]. Proper care must be taken to maintain eyesight immaculately. Immediate medical attention must be given when the eyes are damaged due to any physical or chemical injury. Almost 2.2 billion individuals around the world are affected by vision impairment and blindness [3]. As the WHO reports, one individual loses their eyesight and becomes blind every five seconds [4]. Aging and changes in lifestyle, such as continuous screen time, are the major reasons for impaired vision [2]. Recent research has focused on developing novel therapeutic regimes that can detect and treat different types of ocular diseases such as cataracts, glaucoma and diabetic retinopathy. The specificity and efficacy of current treatment regimes are lowered due to the complex anatomical arrangement and intricate defense mechanism of the human eye [5]. Thus, there is a demand for the development of novel sensors that can be conveniently and comfortably used for the rapid detection and clinical monitoring of ocular diseases.

**Figure 1:**
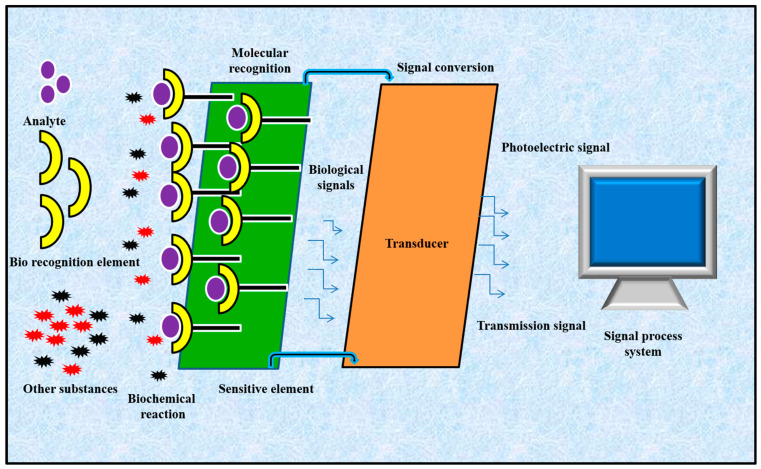
Schematic representation of a biosensor.

Biosensors have gained considerable attention in the field of medicine due to the rising demand for point-of-care diagnostics [6]. They play a vital role in the rapid measurement of biologically important compounds even at minimal concentrations. Biosensors are capable of performing multiple tasks, such as the non-invasive detection of lactic acid, glucose and cortisol in bodily fluids, or tracking essential information about arterial pulse, intracranial pressure, and neural activities [7,8,9,10,11,12,13]. A standard biosensor is composed of two important units: a bioreceptor and a transducer (Figure 1). The bioreceptor can be a nucleic acid or enzyme that can selectively recognize a target analyte, whereas the transducer translates a biorecognition event into a detectable signal that correlates with the amount or presence of a biological or chemical target [14]. Biosensors were primarily fabricated for in vitro quantification of biological samples for single-use home testing or laboratory/point-of-care diagnostics. These devices show promising applications in disease diagnosis due their low cost, high specificity, portability and reduced power requirements. More recently, biosensors have been fabricated as wearable sensors that can effectively sense and monitor body temperature, intraocular pressure, blood pressure, heart rate and oxygen level [15]. New advances in electronics, nanotechnology and microfluidics have led to the development of novel sensors that can offer valuable details about the health status of any individual [16]. There are different types of biosensors: wearable, ingestible and implantable biosensors that are based upon their unique features and applications [17,18]. Wearable biosensors are those which can be worn or connected with the human body for the continuous monitoring of an individual’s actions without disturbing the activity of the user [19]. Wearable skin-patchable sensors are of great interest due to their exceptional role in monitoring sweat composition, heartbeat, body temperature and the movement of various parts of the human body. New advancements in fabricating thin films and flexible electronics, as well as the integration of two or more sensors for developing multisensor devices, have played a vital role in the field of skin-patchable sensors for the real-time monitoring of human health [20]. Implantable biosensors are devices that can be grafted into the human body to obtain precise information about the state of health of a particular organ, such as the heart, bladder, brain and eyes, for treating illnesses such as heart diseases, neurogenic bladder dysfunction, traumatic brain injury and glaucoma, respectively [18,21]. Ingestible sensors or pills are used to detect and monitor the release of therapeutic molecules in a non-invasive method at a specific location inside the human body [22]. 

Biosensors have the ability to diagnose diseases at a preliminary stage and can monitor them continuously. Among the various devices, transducers worn or implanted into eyes have been improved and have had effective clinical applications, which is noteworthy as they have the capacity to directly monitor intraocular pressure (IOP) and humidity on the surface of eyes, and they also offer the option of recording lactic acid, glucose and cortisol levels [16,23,24]. Thus, biosensors serve as a promising platform to obtain imperative details about various disorders such as diabetes, liver and ocular diseases. Among the various ophthalmic disorders, biosensors have been widely used for the early and accurate screening of glaucoma. This review will give detailed information about the use of biosensors in monitoring ocular diseases, especially glaucoma. Nanotechnology-based biosensors for drug delivery and the treatment of glaucoma are also discussed.

## 2. Anatomy of the Human Eye

**Figure 2:**
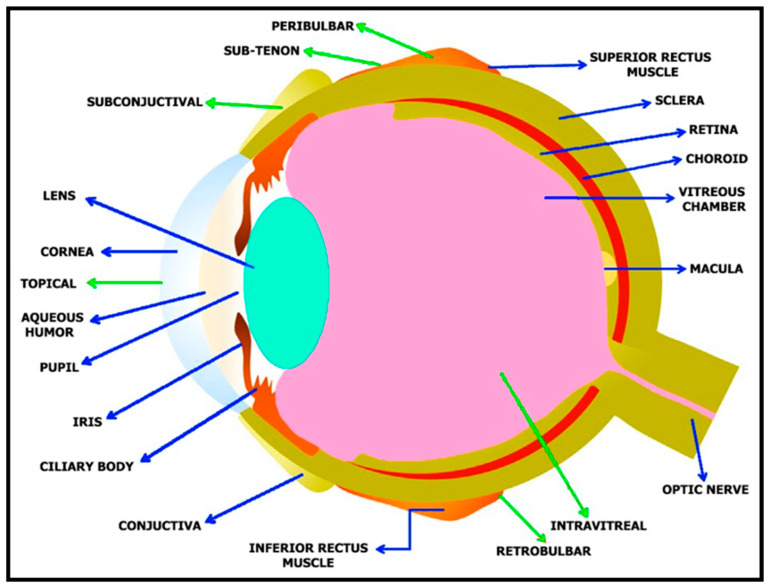
Structure of human eye. Blue arrows denote different structures of human eye and green arrows denote various routes of drug administration. Reprinted from Ref. [29].

The human eye (Figure 2) is globular in structure, with a mass and size of about 7.5 g and 24 mm, respectively [25]. Each ocular tissue possesses a well-defined structure that is responsible for a distinct role in image formation. In humans, the formation of the optic sulcus on the interior surface of the neural ectoderm on either side of the forebrain at three weeks of gestation is the first indication of eye development [26]. The eye contains two important portions, the anterior and posterior segments. The smaller anterior segment collects and focuses light, whereas the larger posterior segment aids in light detection [24]. In the eye, the anterior segment is composed of the conjunctiva, aqueous humor, cornea, ciliary body, lens, and iris, and the posterior segment consists of the choroid, retina, sclera, and vitreous body. Many disorders that impair vision are mainly due to these anterior and posterior segments being affected. These two regions play a vital role in protecting the eye from external foreign bodies [4]. The cornea is a transparent part of the human eye with a thickness of about 0.55 mm at the central region, and a diameter of 11 mm [27]. The conjunctival epithelium, which covers the ocular surface from the limbus to the posterior surface of the eyelids, is composed of a stratified non-keratinized epithelium and goblet cells. The conjunctiva is necessary to maintain the ocular surface’s integrity as it aids in supplying the mucin substance of the tear film and protecting the eye from external stimuli [28]. Light must pass through the aqueous humor in order to reach two important structures, namely, the iris and pupil, which play vital roles in regulating the volume of light passing through the system.

The iris is a pigmented tissue that is situated ahead of the lens and that has the ability to dilate or restrict with the help of specialized muscles, such as dilator and sphincter muscles. With the help of the sphincter muscles, the iris reduces the pupillary aperture under situations of excessive light in an effort to prevent the admission of too much light, as this would otherwise lead to the processing of a muddled blur. When an appropriate volume of light has entered the human eye via pupil, it comes in contact with the lens. The lens is constructed using proteins such as crystallin that are involved in refining the image from the cornea. The lens possesses a higher refraction index than the cornea, owing to its environment requirements. The index of refraction of the lens must be higher than the aqueous humor and vitreous humor for it to further focus the image and support the optical system [30]. The retina’s task is to activate neurons by translating patterns of light stimulus. The retina is found at the rear portion of the eye [31]. Light is absorbed by photoreceptors when it reaches the retinal part of the human eye [2]. Photoreceptors are involved in transforming light energy into neural activity, which is further transmitted to ganglion and bipolar cells. The optic nerve is formed by the axons of ganglion cells that move to the midbrain and thalamic regions, from where the visual information is delivered to the visual cortex. 

**Figure 3:**
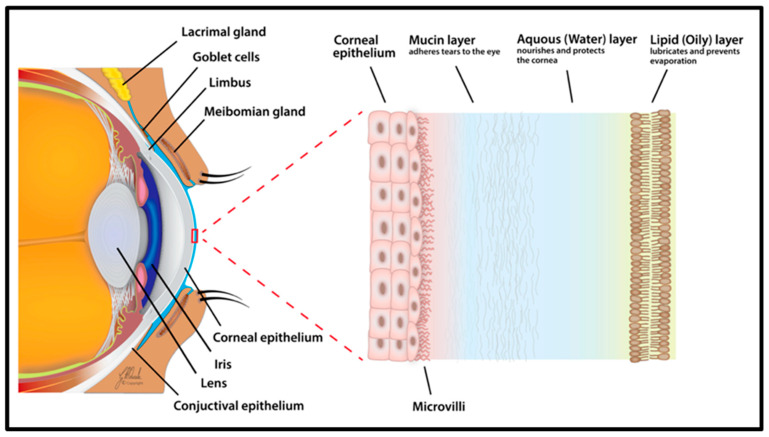
Schematic representation of tear film. The image shows the corneal epithelium, mucin, aqueous and lipid layers of human eye. Reprinted from Ref. [35].

People can view objects that are nearby due to the accommodation process, which enables the human eye to change its optical power by altering the shape of the lens. The refractive power of the eye is dictated by the lens (15–20 diopters), the cornea (40 diopters) and the axial length of the eye (23.3 mm on average). Therefore, the human eye has a convergent refractive power of about 60 diopters. When the refractive power of the cornea, the axial length of the eye and the power of the lens balance out, rays are precisely focused on the retina and emmetropia (perfect vision) is attained [1]. The outer mucosal surfaces of the eye are covered by a special thin fluid layer called the tear film (Figure 3), which is about 3 µm thick and 3 µL in volume [32]. It is a mixture of secretions secreted by several glands situated at the ocular surface, including the lacrimal and meibomian glands. Three layers make up the tear film, each with a unique function and secretion region. The inner layer nearest to the conjunctival epithelium is the mucin layer secreted by the conjunctival goblet cells. The lipid layer of the meibomian glands, which is the outermost oily layer, provides a smooth surface, slows the evaporation of aqueous tears and guards against contamination from external particulates. The lacrimal duct secretes the middle aqueous layer of the tear film, which serves as a lubricant and shields the ocular surface [33]. The tear film, which is frequently referred to as a dynamic (physiological) ocular barrier due to its rapid turnover rate, brief ocular residence time and restricted drug penetration ability, is the first barrier for topically applied drugs [34]. Various factors such as the composition, stability and integrity of the tear film play a vital role in the development of dry eye disease (DED) [35].

## 3. Ocular Diseases

Several ocular tissues are involved in the active functioning of the human eye. Loss of vision has a profound impact on a person’s capacity to preserve their independence and degrades their value of life. Visual impairment is caused by damage to the ocular tissues. Aging and retinal and optic nerve damage stand out as the primary causes of vision loss. The healthcare system is currently facing significant issues as a result of vision impairment [36]. When compared to people who are healthy, those who have ocular disorders have fewer options for employment and higher education, a lower quality of life, and a higher risk of passing away. Approximately 252.6 million people were expected to have moderate to severe visual impairment or to be blind in 2015, which works out to 3.45% of the world’s population (7.33 billion). Vision impairments are now one of the top causes of disability worldwide, and these numbers are still rising quickly. As a result, the World Health Organization (WHO) currently views ocular illnesses as a critical global healthcare issue [37]. Age-related macular degeneration (AMD), cataracts, diabetic retinopathy (DR), glaucoma and dry eye represent common ocular diseases, and their manifestations [38] are described below.

### 3.1. Age-Related Macular Degeneration (AMD)

Elderly people who have AMD may lose their central vision permanently [39]. The most frequent reason for blindness in persons over 50 is AMD, which is characterized by gradual degeneration of retina, choriocapillaris and retinal pigment epithelium (RPE) [40]. Clinical indicators of early-stage AMD include abnormalities in the retinal pigment epithelium. Late AMD causes the loss of central visual acuity, resulting in severe, irreversible vision impairment and eventually leads to a person becoming legally blind, which has a detrimental effect on the affected individual’s life [41]. Even though there are numerous growth factor pathways involved in angiogenesis, VEGF (vascular endothelial growth factor) plays a chief role in the neovascularization of the human eye. Various research reports have concluded that intravitreal injection of VEGF inhibitors can block neovascularization and can be used as an efficient treatment procedure for treating exudative AMD [39].

### 3.2. Cataracts

Cataracts are one of the primary reasons for eye blindness around the world, and are characterized by clouding and blinding one’s vision. The crystalline lens is vulnerable to numerous types of injuries, which can lead to protein misfolding and aggregation, and ultimately result in cataracts. A cataract—a clinical homologue of lens opacity—develops when the lens’ refractive index varies remarkably over the wavelength of transmitted light [42]. The lens is enriched with a combination of both enzymatic and non-enzymatic antioxidants that are actively involved in protecting it by scavenging for reactive oxygen species. When their activity is affected or hindered, this causes damage to the lenticular molecules and results in cataracts.

### 3.3. Diabetic Retinopathy 

Diabetes or an uncontrolled blood sugar level serves as the major reason for the development of diabetic retinopathy (DR). DR damages retinal microvessels gradually and is characterized by microaneurysms, small intraretinal hemorrhages, capillary closures and hard exudates [43]. This leading cause of blindness is linked with excessive angiogenesis, which is a process where new blood vessels are formed from previously existing ones. When there is an increase in the blood sugar level, this results in the damage of blood vessels, causing them to expand, leak and enlarge rapidly. This accelerates neovascularization and leads to the development of diabetic retinopathy [44].

### 3.4. Dry Eye

Keratoconjunctivitis sicca, often referred to as dry eye, arises due to a deficit or the malfunctioning of the tear film. The corneal epithelium begins to pit and decreases the smoothness of the corneal surface. This leads to hazy or cloudy vision. There are two different forms of dry eye, namely, evaporative dry eye or aqueous tear-deficient dry eye. The causative reasons for dry eye are conjunctival scarring due to a deficiency in vitamin A, collagen vascular disorders or adverse effects due to drugs [45]. 

### 3.5. Glaucoma

**Figure 4:**
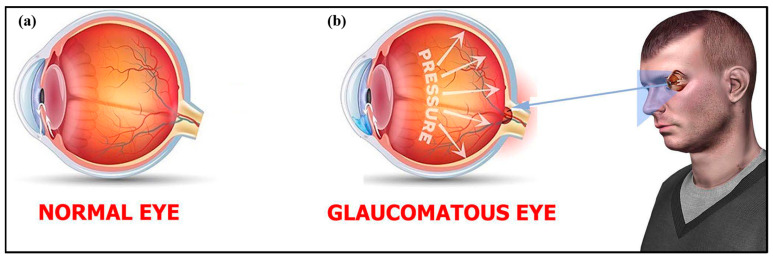
(**a**) Normal eye vs. (**b**) glaucomatous eye. Reprinted from Ref. [29].

**Figure 5:**
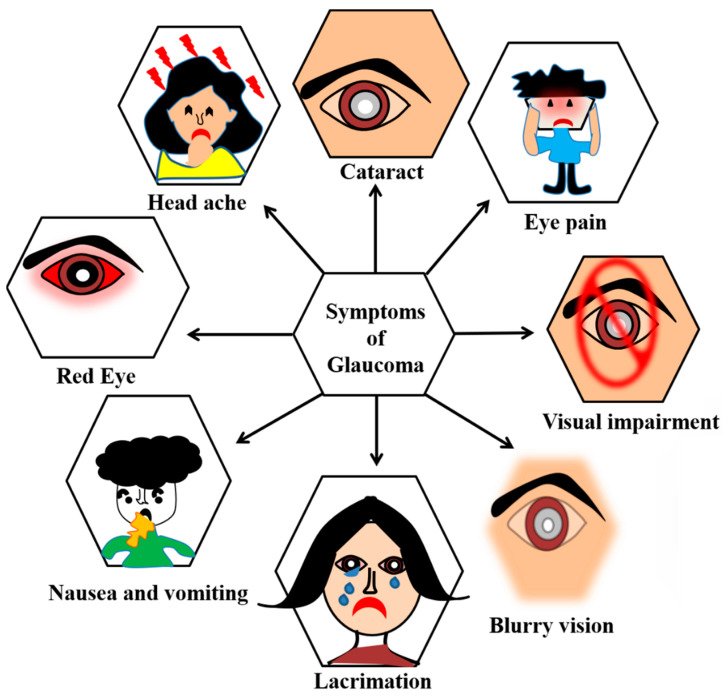
Symptoms of glaucoma include lacrimation, nausea or vomiting, red eye, headache, eye pain, cataract, blurry vision, and visual impairment.

**Figure 6:**
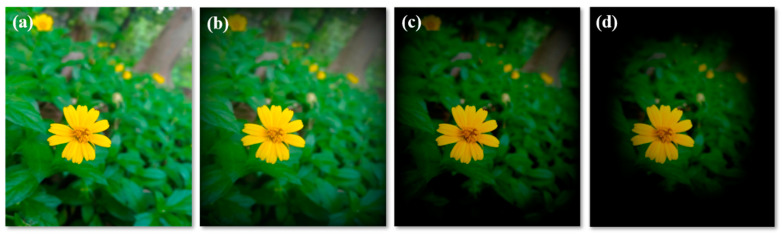
(**a**) Normal vision; (**b**–**d**) early, advanced and extreme stages of glaucoma.

Glaucoma (Figure 4 and Figure 5) is a term given to a group of ocular disorders that is characterized by impaired optic nerves, resulting in progressive loss of vision. The persistent rise in intraocular pressure (IOP) is the most significant hallmark of this disease [46]. Glaucoma is the main reason for blindness among the global population. Increased IOP damages the optic nerves and causes a loss of retinal ganglion cells (RGCs) [47,48]. The three stages of glaucoma are early, advanced and extreme glaucoma (Figure 6). Around the world, 64.3 million people are presently affected, and it is supposed that in 2040 there will be 112 million people suffering from glaucoma [47]. Hence, glaucoma diagnosis and monitoring are very essential, and can be achieved by measuring the IOP [49]. In affected individuals, both retinal ganglion cells and retinal axons become damaged due to high levels of IOP and oxidative stress [50].

## 4. Intraocular Pressure (IOP)

Intraocular pressure (IOP) is an essential measurement that must be recorded during ophthalmic inspections, particularly in patients with ocular hypertension or glaucoma, and in individuals with a high-risk percentage of developing glaucoma [51,52]. Irreversible blindness eventually results from the optic nerve head being damaged by the increase in IOP [53]. Among the various factors such as age, high myopia and family history, IOP is indicated to be the only modifying risk factor for glaucoma. At present, surgery or medication are the only available treatment procedures for glaucoma. Progression of the disease may be reduced by lowering the IOP by thirty to fifty percent using medications. The IOP level rises at night based upon the individual’s circadian rhythm. The IOP values may change even when the eyes are kept closed. These factors significantly contribute to the progression of glaucoma [54]. Normal IOP values fall in between 10 and 22 mmHg, with an average of 15.3 mmHg [55]. Various reports have stated that the IOP level is high in early morning and may be higher during sleeping hours than during the daytime. In addition, sitting posture may also modify IOP measurements.

**Figure 7:**
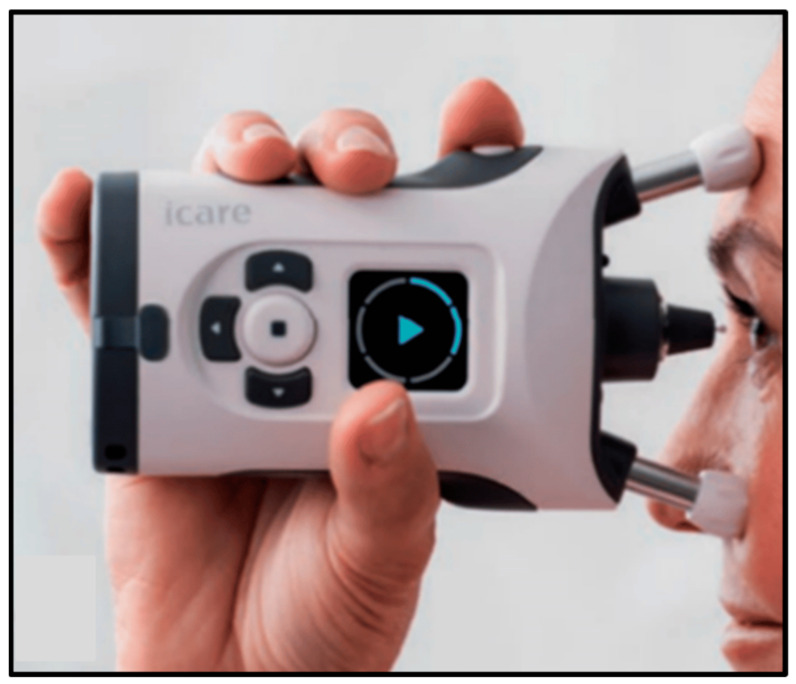
At-home autotonometry can be used by patients at home to measure IOP. Reprinted from Ref. [52].

**Figure 8:**
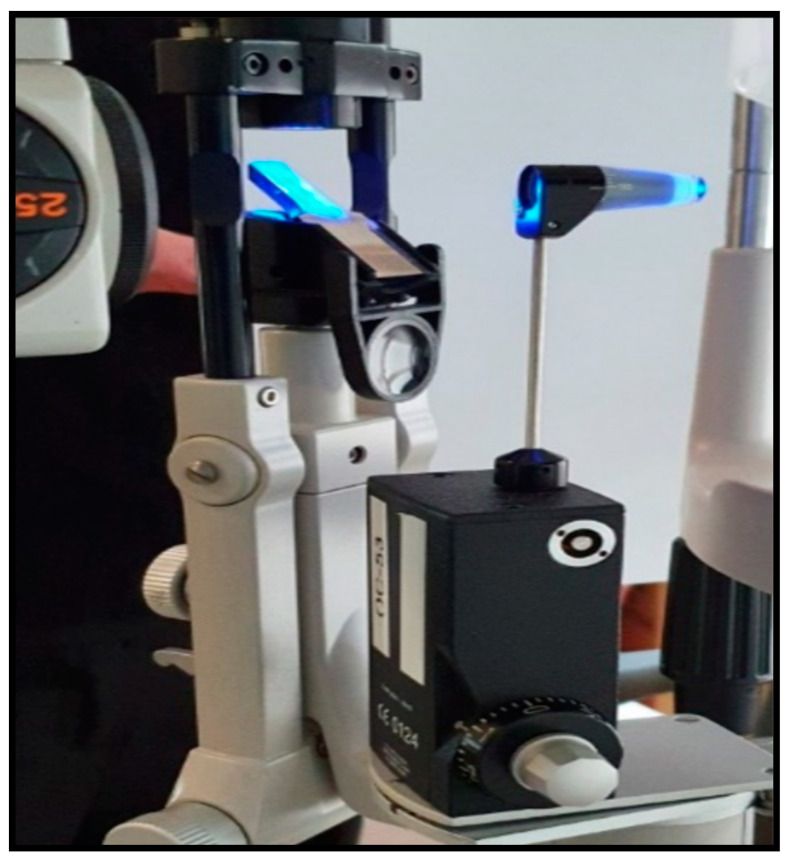
Goldmann applanation tonometry (GAT) is regarded as the “gold standard” for quick and reproducible analysis of IOP. Reprinted from Ref. [52].

**Figure 9:**
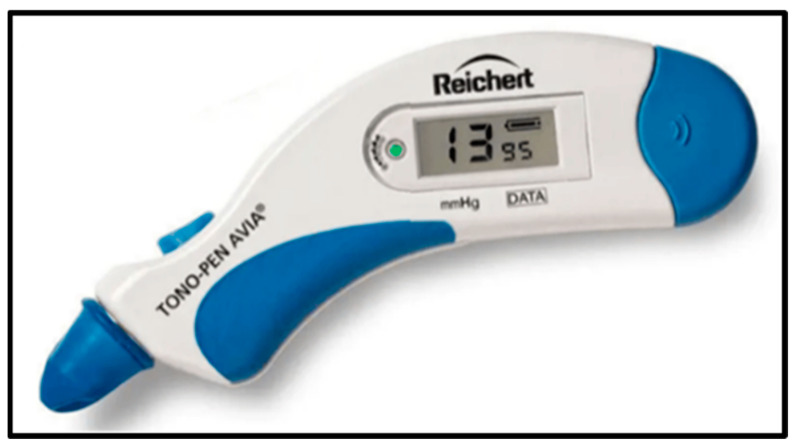
Tonopen is a portable applanation tonometer used for IOP measurement. Reprinted from Ref. [52].

Some of the IOP measuring instruments are applanation tonometers, pneumatonometers, Perkins tonometers, dynamic contour tonometers, wireless implantable transducers, home tonometers and contact lens sensors (Figure 7, Figure 8 and Figure 9) [53]. Goldmann applanation tonometry (GAT) is regarded as the “gold standard” for quick and reproducible analysis of IOP, as it uses the Imbert–Fick equation to calculate IOP. However, GAT requires application of local anesthesia to the corneal eye through hard surface pressing. This method requires the patient to be awakened every 60 min throughout the night to measure the IOP, which further increases the uneasiness among patients. Thus, clinical analysis is not conducive for the early detection, prevention or control of glaucoma. IOP tracking is possible twenty-four hours a day with a wireless implantable transducer (WIT). This method involves wrapping the WIT in an intraocular lens and placing it inside the person (ciliary sulcus) through surgery. A WIT can measure IOP for prolonged time periods to understand the physiological variations in IOP, but it comes with risks of implantation-related infections [53]. A home tonometer is another option to measure IOP. The IOP recorded by a patient at home using home tonometry may vary by 5 mm Hg from the IOP measured by a doctor using a tonometer [54]. A contact lens sensor utilizes the changes in the cornea curvature to attain constant monitoring of IOP. The Tonopen is a portable applanation tonometer, but prior to the analysis local anesthesia and calibration must be performed. Furthermore, the equipment’s limited accuracy reduces its reliability [16]. Several studies have proposed the use of optical sensors to measure pressure, but they still need to deal with the difficulties of implantation and data collection. Implantable sensors can detect changes in IOP without being affected by factors such as blinking, posture and the environment. Unfortunately, the surgical installation of sensors will damage the eyeball and may cause potential side effects. Due to their non-invasive, nearly transparent, highly sensitive and continuous data-recording properties, IOP sensors fabricated using nanoparticles can be efficiently used for IOP detection [56].

## 5. Nanotechnology

**Figure 10:**
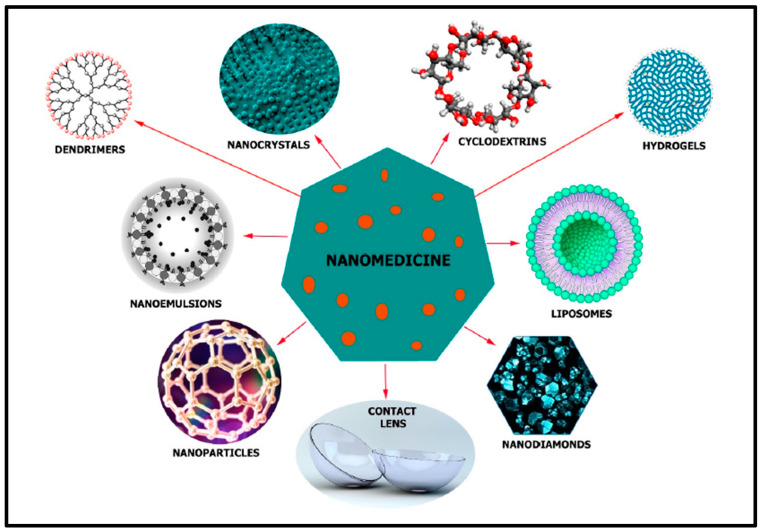
Different types of nanotechnology-based materials that are used for glaucoma treatment. Reprinted from Ref. [29].

Nanotechnology is the development and application of materials (Figure 10), machines or systems with nanometer-scale dimensions [57]. Currently, this area is experiencing rapid expansion within several streams. This technology is expected to achieve greater advancements and play a crucial part in numerous biological applications, including molecular imaging, biosensing drug delivery and disease diagnostics [58]. In the past 20 years, exceptional research has been carried out using nanoscale materials for the betterment of mankind. Nanomaterials are well known for their high surface area, enhanced reactivity, and distinctive mechanical and optoelectronic properties. These properties have been utilized for significant advancements in a variety of industries, including electronics, medicine, food, cosmetics and energy devices [59]. These various advancements in nanotechnology have led to the fabrication of flexible and sensitive nanosensors for biomedical applications. The goal of nanosensors is to identify and quantify any chemical, mechanical and physical alterations linked to a specific marker that plays a vital role in disease development [60]. Graphene is used for biological applications such as biosensing, bioimaging, drug administration, phototherapy and tissue engineering because of its distinctive electrochemical, optical and mechanical capabilities [61]. Mesoporous silica nanoparticles (MSNs) have proven to be highly helpful in a variety of applications, including sensors and catalysis. More significantly, MSNs are used as drug delivering vehicles because of their surface area, surface functionality and biocompatibility [62]. Because of their oxygen permeability, flexibility, transparency, high water content, capacity to load drugs and good biocompatibility, hydrogel-based contact lenses are becoming more popular in the field of ophthalmology [37]. Liposomes are lipid-based spherical vesicles made up of cholesterol and phospholipids. They may be used to encapsulate both hydrophilic and hydrophobic medicines in a single system and are biocompatible, biodegradable and non-toxic. Several efforts have been undertaken to increase the bioavailability, stability, corneal penetration and targeted drug delivery of liposomes [4]. Nanofibers are fibers made of polymers that are in the nanometer size range. Electrospun nanofibers for the regeneration of soft tissues have gained a lot of interest due to their distinctive composition and structural characteristics. The three-dimensional architecture, composition, morphology, biological functions and specific optical, magnetic and electric properties of electrospun nanofibers can be easily controlled based upon the application [63]. A type of molecular aggregation called a polymer micelle is caused by non-covalent bond interactions between several polymer chains. The formation of polymer micelles depends upon specific interactions between the polymers, temperature and pH of the polymer solution used during the synthesis of micelles [64]. Dendrimers are a unique family of macromolecules with a highly branched, three-dimensional architecture and a definite shape and size. They possess a central core and dendritic branches made up of hydrophobic and hydrophilic moieties [65]. The fact that dendrimers may accommodate several molecules inside them or on their surfaces gives them an advantage over linear polymers in synthesizing multifunctional nanoconjugates [66]. The core, dendron and terminal functional groups are the three different components that make up dendrimer molecules [67]. The remarkable electrical and optical properties of gold and other noble metal nanoparticles (NPs) are used for different types of applications in the field of biomedicine, such as molecular disease diagnosis/imaging, drug delivery and the development of biosensors. Contact lenses loaded with gold nanoparticles were used to study their role in improving the amount of the drug timolol, which is widely used for treating glaucoma [68]. 

## 6. IOP Monitoring with Nano-Based Sensors

IOP-lowering treatments are beneficial for most of the people with glaucoma since an elevated IOP is known to be a main risk factor for optic nerve injury. First-line glaucoma medication normally begins with eye drops that reduce IOP through one of two main mechanisms: increasing the aqueous humor outflow through the trabecular or uveoscleral pathways, or by suppressing aqueous humor production. When topical medicines are ineffective at reducing IOP, laser therapy or surgery may be performed. Brimonidine, brinzolamide, bimatoprost, carteolol, latanoprost, pilocarpine, timolol and travoprost are a few of the medications that have been studied as nanomedicine formulations for the treatment of glaucoma [69]. Table 1 depicts the different types of sensors for the efficient monitoring of IOP in eyes.

Liao et al. fabricated pilocarpine-loaded gelatin MSNs with continuous-release characteristics for the intracameral pharmacotherapy of glaucoma. The prepared material was intracamerally injected into the anterior chamber to decrease the level of IOP. A fifty percentage pilocarpine release was observed for a period of thirty-six days. In vivo experiments proved the maintenance of intraocular pressure in eyes with ocular hypertension for a period of twenty-one days [62]. 

A highly reliable and low-cost method to develop the graphene grating on contact lenses was first proposed and demonstrated by Tang et al., who carried out direct laser interference patterning of graphene film on commercial contact lenses using a Nd:YAG laser. During the fabrication process, two different features—film thickness and interference angle—were taken into consideration. The diffraction pattern of the graphene grating fabricated on the contact lens played a vital role in monitoring the normal functioning of the eye. Any change in the shape or bending of the contact lens can be recorded through the alterations in the diffraction pattern. The prepared graphene film on the contact lens proved to be flexible and conductive, and may serve as a suitable platform for designing a circuit for the construction of smart contact lenses [70].

The conventional method of using eye drops for treating glaucoma has numerous disadvantages such as poor drug bioavailability owing to the quick clearance of the drops from the preocular space. Kim et al. prepared amino-functionalized mesoporous silica (AMS) particles and studied their role as delivering vehicles for the drug brimonidine. In total, 41.17 μg/mg of brimonidine was loaded inside the particles with a sustained drug release for up to eight hours. The presence of hydroxyl and amino groups made the prepared particles mucoadhesive, and hence, when applied topically onto the eyes of rabbits, the brimonidine–AMS particles were able to stay in the preocular space for up to twelve hours. The efficacy of the prepared brimonidine–AMS particles in reducing the intraocular pressure was analyzed using an in vivo model [71].

The treatment of glaucoma involves multiple drugs, among which a combination of timolol maleate and brimonidine tartrate is commonly used to treat the disease. Timolol-maleate–brimonidine-tartrate-loaded liposomes were prepared using a thin-layer hydration method. Eight different formulations were prepared by Bigdeli et al. using the timolol-maleate–brimonidine-tartrate-loaded liposomes. Among the eight formulations, one formulation was used for in vivo experiments based upon the primary evaluations. The efficacy of the prepared formulation in reducing intraocular pressure was evaluated using an in vivo model, which demonstrated its potential application in the field of glaucoma treatment. The results showed that the formulation has potential therapeutic efficacy in lowering the IOP level and in helping the IOP level return to normal after ninety hours [72].

Gagandeep et al. utilized an electrospinning method to fabricate nanofibers loaded with timolol maleate and dorzolamide hydrochloride for the efficient treatment of glaucoma. The nanofibers had a mucoadhesive property and were able to show sustained release of the drug for a period of up to twenty-four hours. In vivo experiments carried out by inserting the nanofiber patch into the cul-de-sac areas of glaucoma-induced rabbits showed a significant reduction in the IOP level when compared with conventional eye drops [73].

Xu et al. fabricated a novel method where timolol and latanoprost were loaded inside a micelles-laden contact lens for treating glaucoma. The formulation was prepared by free-radical polymerization of a 2-hydroxyethyl methacrylate monomer. and the drugs timolol and latanoprost were loaded within mPEG-PLA micelles. The prepared formulation showed sustained drug release of timolol and latanoprost for a period of 144 h and 120 h, respectively, in a simulated tear fluid. When compared with eye drops, the formulation improved the mean residence time and bioavailability of both drugs. In vivo experiments using a rabbit model showed a reduction in IOP level for over 168 h. Thus, the prepared micelles-laden contact lens can be efficiently used to deliver two different drugs for a long time period for glaucoma treatment [74].

Wang et al. designed dendrimer gel particles that possess the advantages of dendrimers, hydrogels and particles. Dendrimer gel particles were fabricated using an inverse emulsion technique in combination with the aza-Michael addition reaction method. Two different drugs, namely, brimonidine tartrate and timolol maleate, were loaded inside the dendrimer gel particles to study their drug-delivering efficacy. Non-toxicity, corneal permeability, degradability and drug release kinetics were also studied, which proved their efficacy in treating glaucoma [75].

Sonntag et al. used gold nanoparticles and studied their role as delivering vehicles of antiglaucoma drugs. The optimum size for targeting the trabecular meshwork was studied using different-sized gold nanoparticles (5, 60, 80 and 120 nm) that were either bare gold nanoparticles or those coated with hyaluronic acid. The colloidal stability, in vitro cellular uptake and ex vivo distribution in the anterior chamber of the eye was studied for bare and hyaluronic-acid-coated gold nanoparticles. The coating of hyaluronic acid avoided the aggregation of gold nanoparticles inside the trabecular meshwork even after applying them to the porcine eye. Nanoparticles that were 120 nm in size were seen to be accumulated at the higher concentration in the trabecular meshwork [76].

Vandamme et al. studied the role of size, molecular weight, number of hydroxyls, and amine and carboxylate surface groups in different poly(amidoamine) dendrimers for the controlled release of drugs. A single dose of dendrimer solution was applied onto the eyes of albino rabbits to study the ocular tolerance and retention time of the poly(amidoamine) dendrimer formulations. Dendrimers with hydroxyl and carboxylic surface groups possessed a longer residence time. The remanence time was dependent upon the size and molecular weight of the poly(amidoamine) dendrimer solution [77]. 

Maulvi et al. studied the role of gold nanoparticles in the uptake of the antiglaucoma drug timolol. The conventional soaking method which is widely used to load timolol did not affect the properties of the contact lens, but resulted in disadvantages such as poor drug loading and increased burst release of loaded drugs. Two different approaches were used: (i) gold nanoparticles were loaded into the timolol soaking solution and (ii) contact lenses were incorporated with gold nanoparticles. Further, the contact lenses were soaked in various concentrations of timolol (2 and 4 mg/mL). Optical transparency and swelling behavior were not affected due to the presence of gold nanoparticles. Both of these approaches did not show any significant improvement in the release behavior of timolol. In vivo experiments proved that there was a 2 mmHg average fall in IOP (72 h) in the groups treated with gold-nanoparticle-laden contact lenses, whereas soaked contact lenses without gold nanoparticles and conventional eye drops were measured to be 2 mmHg. A significant improvement in the deposition of drugs in the ciliary muscles and conjunctiva was observed with gold-nanoparticle-laden contact lenses, thereby proving their efficacy in treating glaucoma [78].

Wu et al. proposed the loading of brinzolamide into liquid crystalline nanoparticles and studied their role in treating glaucoma. A modified emulsification technique was used for preparing the drug-loaded nanoparticles. When compared with the brinzolamide commercial product, brinzolamide-loaded liquid crystalline nanoparticles showed prolonged drug release. The ex vivo apparent permeability coefficient of brinzolamide loaded into liquid crystalline nanoparticles showed a 3.47-fold increase when compared with the brinzolamide commercial product. In vivo experiments proved that the brinzolamide-loaded liquid crystalline nanoparticles can serve as efficient delivering vehicles for treating glaucoma with improved ocular bioavailability and lower concentrations of drugs [79]. 

**Figure 11:**
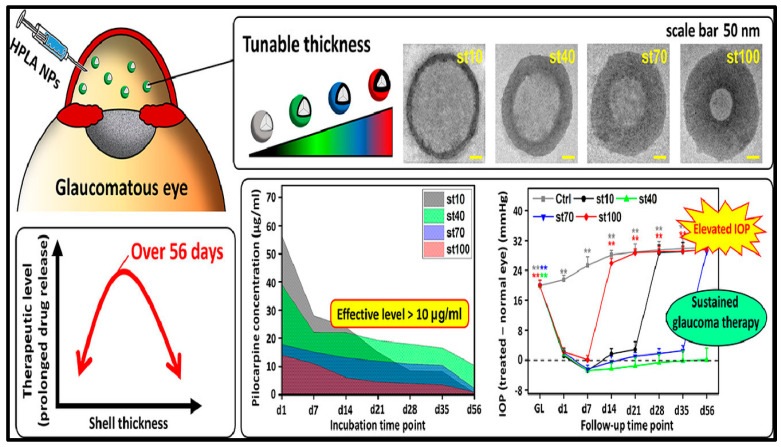
Poly (lactic acid) based intraocular drug delivery systems for treating glaucoma. Asterisks denote statistically significant differences (** *p* < 0.005) as compared with the baseline IOP values. Reprinted with permission from [80].

Nguyen et al. prepared hollow poly (lactic acid) nanoparticles and studied the role of shell thickness in the sustained release of antiglaucoma drugs (Figure 11). Pilocarpine-loaded hollow poly (lactic acid) nanoparticles with a thickness from about 10 to 100 nm proved to be non-toxic to human lens epithelial cells and rabbit eyes. Thick shells from 70 to 100 nm showed sustained release of the drug pilocarpine with reduced drug loading efficacy. Moderately thick shells with a thickness of about 40 nm showed sustained release of pilocarpine for up to fifty-six days. This formulation also proved to alleviate ocular hypertension for over fifty-six days when tested on an in vivo rabbit model. The results demonstrated the role of shell thickness in the long-term delivery of antiglaucoma drugs [80].

**Table 1:** Different types of biosensors fabricated for efficient monitoring of intraocular pressure.

Key Materials	Fabrication	Model Used for the Study	Ref.
Polydimethylsiloxane (PDMS) and polyurethane-based elastomers	Soft lithography	Enucleated porcine eye	[23]
Polydimethylsiloxane (PDMS), parylene C	Soft lithography	Porcine eye	[24]
Silver nanowires	Spin coating, photolithography, wet etching	In vitro: polydimethylsiloxane model eye; in vivo: rabbit eye	[55]
Graphene, polydimethylsiloxane (PDMS), parylene C	Chemical vapor deposition (CVD)	Silicone eyeball	[56]
Graphene nanowalls, polydimethylsiloxane (PDMS), silver wires	Plasma-assisted chemical vapor deposition (PACVD), spin coating	Porcine eye	[81]
Si-nanomembrane, Au-loaded coil, Cu-inductive coil	3D printer, spinning, Photolithography	Rat eye	[82]
Monodisperse silica nanoparticles, gold nanobowl (AuNB) substrate	Hydrofluoric acid (HF) etching technique	Porcine eye	[83]
Platinum-stain gauge, polyimide, polydimethylsiloxane (PDMS)	MEMS (microelectromechanical system) process, spin coating	Silicone eyeball	[84]
Gold hollow nanowires	Spin coating	Rabbit eye	[85]
Polydimethylsiloxane (PDMS)	Casting process with the molds manufactured with a high-speed micromilling machine	Enucleated porcine eye	[86]
Polydimethylsiloxane (PDMS), polystyrene	Spin coating	In vitro: artificial silicone eye model; ex vivo: porcine eyeball	[87]
Polydimethylsiloxane (PDMS), polyethylene terephthalate (PET)	Chemical-assisted bonding and thermoforming technologies	Porcine eye	[88]
Silver conductive paint	Painting	Finite-element based model	[89]
Polydimethylsiloxane (PDMS)	Casting method	Enucleated porcine eyes	[90]
Nanostructured Si_3_N_4_-membrane, poly(methyl methacrylate) (PMMA) and polystyrene	Bottom-up fabrication process based on polymer phase separation, spin coating, E-Beam evaporation, Reactive Ion etching	Rabbit eye	[91]
Silicon dioxide (SiO_2_), silicon nitride (SiN), Al_2_O_3_ layer	Low-pressure chemical vapor-deposition (LPCVD), reactive ion etching, photolithography, electron beam lithography	Rabbit eye	[92]
Graphene	Chemical vapor deposition (CVD)	Porcine eye	[93]
Graphene–silver nanowires (AgNW), polyethylene terephthalate and polydimethylsiloxane (PDMS), PMMA	Spin coating, CVD method, etching, photolithography	Bovine eye	[94]
Silicon wafer, ferrite, PDMS	Etching, coating	Rabbit eye	[95]
Poly-2-hydroxyethyl methacrylate (poly HEMA), parylene C	Cast-molding method	Artificial anterior chamber	[96]
SU-8 photoresist, AZ9260 resin, Copper	Spin coating, sputtering, Lithography, etching	Silicone Rubber model eye	[97]
Liquid silicone elastomer, copper foil	Molding, etching	Porcine eye	[98]
PDMS, copper, gold, titanium, parylene C	Chemical vapor deposition (CVD), plasma etching, UV lithography	Canine eye	[99]
SiN, Titanium, polyimide, Titanium/Copper	Low-pressure chemical vapor deposition (LPCVD), sputtering, spin coating, photolithography	Rabbit eye	[100]

Agaoglu et al. fabricated a microfluidic-based sensor that could convert minute strain changes into a large fluidic volume expansion that can be easily detected using the camera of smart mobile phones. The fabricated sensor had a detection limit of <0.06% for uniaxial strain and <0.004% for biaxial strain. Further, the sensor was embedded in a silicone contact lens and IOP-induced strain was measured in the physiological range in porcine eyes. The sensor can continuously operate for more than nineteen hours with the maximum life time of about seven months, which proves its role in ophthalmology [23]. 

**Figure 12:**
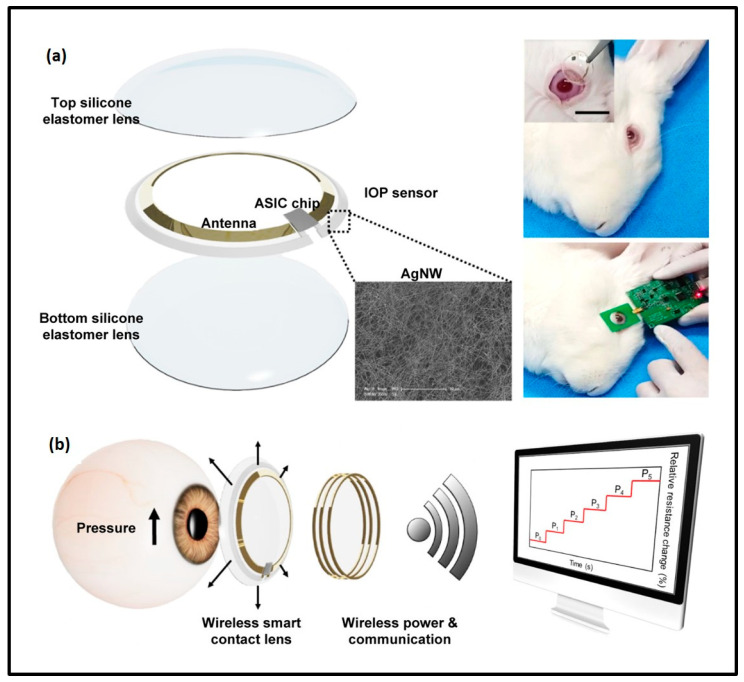
Schematic representation of a wireless smart contact lens. (**a**) Smart contact lens is placed on a rabbit’s eye. (**b**) IOP monitoring using the smart contact lens. Reprinted with permission from [55].

Kim et al. proposed a non-invasive method for the continuous monitoring of IOP using smart contact lenses integrated with transparent silver nanowires, IOP strain sensors and wireless circuits. The robust stability of the fabricated IOP sensors within the smart contact lens was analyzed in the presence of tears and with the eyelid blink model. In vivo experiments using a rabbit eye model proved the efficacy of the integrated wireless smart contact lens in the continuous monitoring of IOP, and thereby proved the method’s role in the management of glaucoma (Figure 12) [55].

Xu et al. constructed a transparent, highly sensitive, biocompatible, graphene-based, non-invasive sensor for the continuous monitoring of IOP. The sensitivity and accuracy of the sensor was improved using a graphene Wheatstone bridge consisting of two strain gauges and two compensating resistors. When tested on a silicone eyeball, the voltage output was proportional to the IOP fluctuation, thereby proving its efficacy. A wireless system was designed for the sensor to monitor IOP using a mobile phone [56].

Liu et al. developed a non-invasive method to continuously monitor IOP using a new strain gauge material based upon graphene nanowalls. The association between corneal strain, contact lens and IOP was determined using stimulation. A novel method was designed to transfer graphene nanowalls onto the contact lens with the use of a gold film for non-invasive monitoring of IOP. The device had an IOP detection sensitivity of 42,250 ppm/mmHg, which is higher than that of the IOP tonometer [81].

Kim et al. fabricated a smart contact lens for monitoring IOP. A strain sensor was placed inside the lens which can perfectly monitor IOP. The contact lens also has the ability to transmit the IOP values using an antenna. The IOP values obtained using the smart contact lens were comparable with those measured using a tonometer, which proves their efficacy in monitoring IOP in diabetic individuals undergoing intraocular islet transplantation [82].

**Figure 13:**
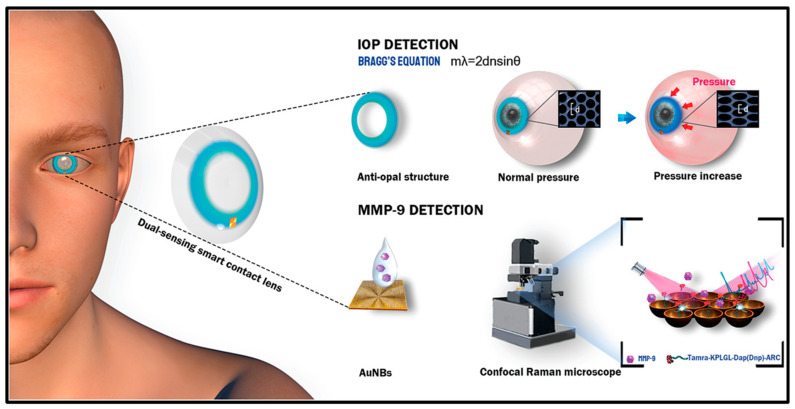
Contact lens sensor for IOP monitoring and MMP-9 detection. Reprinted from [83].

Ye et al. fabricated a smart contact lens sensor that can be efficiently used for monitoring IOP and detecting matrix metalloproteinase-9 (MMP-9). Both of these biomarkers serve as important indicators for various ocular diseases, especially glaucoma. The contact lens sensor was able to continuously monitor higher levels of IOP by applying an antiopal structure that could detect changes in color without the aid of electronic systems. The sensor was able to quantify nanomolars of MMP-9 in real tear samples (Figure 13) [83]. 

## 7. Future Perspectives 

Biosensors play a vital role in the field of point-of-care disease diagnostics [101]. Until now, several research reports have shown that increased IOP is well correlated with various diseases such as obesity, diabetes, retinal vein occlusion and hypertension. However, its mechanism and association with diseases still remain uncertain and controversial [16]. Moreover, the IOP is not the same in all parts of the eye. The major problems encountered in fabricating IOP sensors are as follows: an IOP biosensor must be safe, accurate, biocompatible and reproducible, and must easily allow for self-measurements by the user; additionally, the sensor developed must mimic the equipment used in the hospital in terms of IOP measurement results. The potency of IOP sensors fabricated in the laboratory has been tested using model or animal eyes instead of human eyes. Some sensors hold a reading distance of about 5 mm. However, the eyelashes of most people can grow up to 8–12 mm, and thus the distance of the sensor must be less than 8 mm from human eyes in order to evade any interactions with the eyelashes [102,103,104]. The key factors to be studied during the preparation of IOP sensors are long term stability and biocompatibility, because the materials may fail or become damaged due to continuous mechanical deformation. This will, in turn, reduce the performance of the sensor when used for continuous or long-term monitoring of IOP. Transparent IOP sensors with good flexibility can be prepared using conductive materials to realize energy and data transmission. Moreover, transparent IOP sensors that are miniature in size and ultra-thin can be convenient for patients to use continuously [53]. Moreover, good water content and wettability are two important features that must also be considered when fabricating a wearable IOP sensor. Generally, polymerized hydroxyethyl methacrylate (pHEMA) and silicone hydrogel (SiH) are widely used for preparing commercial contact lenses. However, these lenses are susceptible to hydration and may result in disadvantageous noise while monitoring the IOP [105,106,107]. Rubber and PET are also used for the fabrication of contact lenses as they are easily scalable and less vulnerable to the changes in environmental hydration [108,109]; however, the reduced transmission of oxygen, unacceptable hardness and improper water content are the major drawbacks associated with these materials [110]. The details received from the IOP monitoring sensors can be used to prevent additional complications of the disease. They can also be used to understand the circadian rhythm variations in IOP. IOP biosensors will help clinicians and researchers to address the main problems in treating different kinds of ocular disorders. More research must be focused on developing IOP sensors with integrated systems that can offer valuable information about the stage of glaucoma for proper treatment. 

## 8. Conclusions

Glaucoma is a term given to a group of ocular disorders that is characterized by impaired optic nerves, resulting in progressive loss of vision. The persistent rise in IOP is the main cause of the disease. Although there are multiple approaches available to monitor IOP, a novel non-invasive, transparent, highly sensitive, IOP biosensor is needed to overcome the drawbacks associated with current diagnostic procedures. Wearable and implantable biosensors fabricated using nanoparticles and integrated systems can be preferably be used due to their promising role in the continuous monitoring of IOP. Currently, few clinical trials have been carried out for the constant monitoring of IOP using implantable sensors. In the future, smart contact lenses with IOP sensors can serve as an efficient strategy to detect and treat glaucoma. However, they must be put into clinical practice for early diagnosis and timely treatment.

## Data Availability

Not applicable.
